# Supplementary material for: The Systems Biology Research Tool: evolvable open-source software
Source: BMC Syst Biol. 2008 Jun 29;2:55. doi: 10.1186/1752-0509-2-55 (PMC2446383; doi:10.1186/1752-0509-2-55)
Supplement: Additional file 1 — SBRT Archive. An archive of the current version of the Systems Biology Research Tool. [file 1752-0509-2-55-S1.zip › sbrt-1.4.0/doc/developers_guide/api/sbrt/kernel/fba/comp/FbaSolver.html]

FbaSolver


|  |  |  |
| --- | --- | --- |
| **Overview** \| **Package** \| **Class** \| **Use** \| **Tree** \| **Deprecated** \| **Index** \| **Help** | |  |
| **PREV CLASS**   **NEXT CLASS** | **FRAMES**    **NO FRAMES**     **All Classes** |  |
| SUMMARY: NESTED \| FIELD \| CONSTR \| METHOD | DETAIL: FIELD \| CONSTR \| METHOD |  |


---


## sbrt.kernel.fba.comp Class FbaSolver

```
java.lang.Object
  sbrt.kernel.fba.comp.FbaSolver
```

---

``` public class FbaSolver extends java.lang.Object ```

This class is used to solve the system of linear equations represented by
*Sv = 0*.

**Author:**
:   This class was written and documented by Jeremiah Wright
    while in the Wagner lab.

---

**Constructor Summary**

`FbaSolver()`


| **Method Summary** | |
| --- | --- |
| `static LinearSystemSolution<java.lang.String>` | `solve(Fluxome fluxome, LinearSystemSolver<java.lang.String> solver)`             Solves *Sv = 0*, where *S* is the stoichiometry matrix of the provided fluxome and *v* is the vector of fluxes.** |

**Methods inherited from class java.lang.Object**

`clone, equals, finalize, getClass, hashCode, notify, notifyAll, toString, wait, wait, wait`

**Constructor Detail**

### FbaSolver

```
public FbaSolver()
```


**Method Detail**

### solve

```
public static LinearSystemSolution<java.lang.String> solve(Fluxome fluxome,
                                                           LinearSystemSolver<java.lang.String> solver)
```

:   Solves *Sv = 0*, where *S* is the stoichiometry matrix of the provided
    fluxome and *v* is the vector of fluxes.

    :   **Parameters:**: `fluxome` - the fluxome used to define Sv = 0.: `solver` - the linear system solver to be used. **Returns:**: a solution to the system of linear equations represented as Sv = 0.**


---


|  |  |  |
| --- | --- | --- |
| **Overview** \| **Package** \| **Class** \| **Use** \| **Tree** \| **Deprecated** \| **Index** \| **Help** | |  |
| **PREV CLASS**   **NEXT CLASS** | **FRAMES**    **NO FRAMES**     **All Classes** |  |
| SUMMARY: NESTED \| FIELD \| CONSTR \| METHOD | DETAIL: FIELD \| CONSTR \| METHOD |  |


---
